# Characterization of infectious laryngotracheitis virus isolated from commercial layer chickens in Bangladesh during the year 2021–2022

**DOI:** 10.5455/javar.2024.k789

**Published:** 2024-06-09

**Authors:** Md. Mostofa Kamal, Mohammad Sadekuzzaman, Kohinoor Parvin, Md. Enamul Haque, Sajedul Hayat, Md. Ariful Islam, Mst. Minara Khatun, Mahbubul Pratik Siddique, Sham Soun Nahar, A. K. M. Khasruzzaman, Muhammud Tofazzal Hossain, Md. Alimul Islam

**Affiliations:** 1 Department of Microbiology and Hygiene, Bangladesh Agricultural University, Mymensingh, Bangladesh; 2 Central Disease Investigation Laboratory, Department of Livestock Services, Dhaka, Bangladesh; 3 Department of Microbiology, Sheikh Hasina University of Science and Technology, Bhairab, Bangladesh; 4 Livestock Research Institute, Mohakhali, Dhaka, Bangladesh

**Keywords:** Layer chickens, PCR, AGIDT, VNT, Mice, ITPI

## Abstract

**Objective:**

Infectious laryngotracheitis virus (ILTV) is responsible for causing infectious laryngotracheitis (ILT), which is a rapidly spreading and extremely transmissible disease in chickens. The current research aims to isolate and characterize ILTV from layer chickens in Bangladesh.

**Materials and Methods:**

A total of 345 samples (trachea, larynx, and lungs) were collected from ILT-suspected dead and sick layer chickens of 32 ILT-suspected farms in three different outbreak districts (Gazipur, Tangail, and Mymensingh) of Bangladesh during the outbreak year 2021-2022. Rapid detection kits examined the samples for avian influenza virus (AIV) and Newcastle disease virus (NDV). ILTV-specific primers were used to screen 72 NDV- and AIV-negative samples by polymerase chain reaction (PCR). Using chorioallantoic membrane (CAM), the study isolated the ILT virus from 9 to 10-day-old seronegative embryonated chicken eggs (ECEs) using selected PCR-positive samples. The virus was confirmed using nucleotide sequencing, agar gel immunodiffusion test (AGIDT), viral neutralization test (VNT), and pathogenicity evaluations using mortality index for chicken embryos (MICEs) and intra-tracheal pathogenicity index (ITPI).

**Results:**

The results indicated that among the PCR-positive 10 samples, only two (Alim_ILT_1001 and Alim_ILT_1,000) were found positive using ECEs. There were two field isolates of ILTVs, as shown by the amplicon size of the ICP4 gene-based PCR. A phylogenetic study of the ICP4 gene revealed that the recent isolates have a close similarity with the ILTV isolates of Turkey, Bangladesh, and Australia. AGIDT revealed strong precipitation lines due to ILTV-specific antibodies reacting with field viruses, while VNT neutralized both isolates with conventional ILTV antibodies. The pathogenicity testing indicated that Alim_ILT_1001 had MICE and ITPI values of 0.77 and 0.63, whereas Alim_ILT_1,000 had 0.71 and 0.57.

**Conclusion:**

Both the ILTV isolates have similarities with the isolates of Turkey, Bangladesh, and Australia, and they are highly virulent for chickens.

## Introduction

The poultry industry is a significant sector in Bangladesh’s agriculture, playing a crucial role in providing a substantial portion of the country’s animal protein supply, including white meats and eggs [1]. Infectious laryngotracheitis (ILT) is a notable viral disease that specifically targets the upper respiratory system of chickens. The disease is caused by a specific virus called *Gallid herpes virus-1*, which is classified under the Herpesviridae family, Alphaherpesvirinae subfamily, and Iltovirus genus [2,3]. Chickens serve as the main natural host, and the most distinctive indications are detected in mature birds [4]. Chickens get Infectious laryngotracheitis virus (ILTV) through the upper nasal and intraocular routes [5]. Ingestion may provide an alternative route for infection, but further exposure to the nasal epithelium is necessary. A higher rate of transmission happens in birds that are currently afflicted [6]. The symptoms in severe cases are characterized by gasping, coughing, the expulsion of bloody mucus, and significant difficulty breathing, which can potentially result in asphyxia [2,7,8].

Depending on the immunological condition of the flock and the virulence of the strain, ILT can cause a mortality rate of 100%. Additionally, the fatality rate can surpass 50% [9]. The ILT virus can result in significant mortality and reduced egg production in both broiler and layer hens [10].

Although ILTV strains demonstrate antigenic homogeneity, there is natural variation in the virulence of ILT virus strains. Some strains are very virulent, resulting in significant morbidity and mortality, while others have low virulence and only cause mild or undetectable infections [11]. Furthermore, the disease is prevalent in regions with concentrated chicken farming, and its occurrences lead to significant financial losses as a result of increased death rates, reduced growth rates, and diminished egg production [12]. The initial recorded incidence of ILTV infection was detected in the United States in 1925 [2].

Numerous countries in Asia, Europe, South America, North America, and Asia have since confirmed the presence of the virus. Islam et al. [13] reported the first occurrences of the disease in Bangladesh. Jahan et al. [14] and Rahman et al. [11] performed an enzyme linked immunosorbent assay (ELISA) test to detect the seroprevalence of ILT infection in commercial chickens from different areas of Bangladesh.

The ILTV was isolated by cultivating seronegative embryonated chicken eggs (ECEs) using the chorioallantoic membrane (CAM) route. Moreover, avian cell cultures such as chicken embryo liver (CEL), chicken embryo kidney (CEK), chicken kidney (CK), and chicken embryo liver (CELu) can also be used for the isolation of ILTV [2,13]. AGDT, VNT, passive hemagglutination (PHA), ELISA, and indirect immunofluorescence assays effectively identify antibodies against ILTV [10]. The ILTV can be detected using both conventional and real-time polymerase chain reaction techniques [15,16]. Determining the specific type of ILTV present in the field or causing clinical outbreaks is challenging due to the significant antigenic and genetic similarities among ILT viruses [17].

Numerous investigators investigated the prevalence and incidence of ILTV, as well as its pathophysiology, immunity, propagation, and diagnostics [18,13]. Considering the frequent incidence of transmitted diseases and the subsequent economic damages for poultry producers, it is widely recognized that there is an urgent national need to identify and protect the chicken population from such diseases swiftly. Poultry breeders in Bangladesh are utilizing imported ILT vaccinations to control ILT without considering the local isolates or serotypes of ILTV. Bangladeshi poultry farmers are using commercially marketed ILT vaccinations to immunize their chickens against ILTV without assessing the pathogenicity of the disease.

This study aimed to isolate and characterize ILTV from clinical samples of ILT-susceptible commercial layer chickens from different outbreak areas of Bangladesh by using embryonated chicken eggs that were negative for the virus, as well as serological tests (AGIDT and VNT), pathogenicity tests mortality index for chicken embryos (MICE and intra-tracheal pathogenicity index (ITPI), and PCR and nucleotide sequences of the ICP4 genes of the viruses to find out where the circulating strains of ILTV came from.

## Materials and Methods

### Ethical statement

The research was performed under the guidelines of the Animal Welfare and Experimentation Ethics Committee (AWEEC) at Bangladesh Agriculture University in Mymensingh. The document was granted official sanction with the reference number [AWEEC/BAU/2021 (53)].

### Sample collection and screening with a rapid Newcastle diseases virus (NDV)/ avian influenza virus (AIV) test kit 

**Figure 1.**
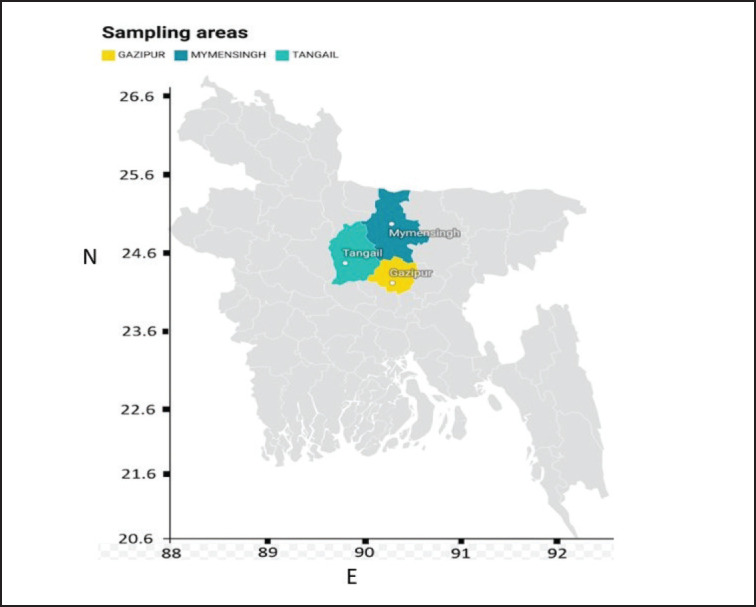
Map showing different districts of sampling.

A total of 345 samples (trachea, larynx, and lungs) were collected from 32 suspected commercial layer farms in different outbreak areas of Bangladesh, such as Tangail, Mymensingh, and Gazipur districts (Fig. 1). The birds showed a drop-in egg production, significant respiratory symptoms, and comparatively high rates of morbidity and mortality. Not one of these flocks received an ILTV vaccination. The time frame for collecting these samples was November 2021–December 2022. Following an initial screening using the rapid test kits for AIV and NDV (Anigen Rapid AIV Ag/NDV Ag test kit, USA), the samples were taken in isotonic phosphate-buffered saline (1XPBS) followed by a manufactured protocol. The samples were stored in an icebox throughout transportation to the virology laboratory of the Department of Microbiology and Hygiene at BAU in Mymensingh. Once the samples arrived at the lab, they were immediately stored at −80°C until they were processed. Among the 345 samples, 72 were found negative for NDV/AIV by using a rapid kit, which was used for further screening of the ILT virus by PCR.

### Screening samples for ILTV by PCR

The deoxyribonucleic acid (DNA) from 72 suspected ILTV field samples was isolated using the QIAamp DNA Micro Kit following guidelines provided by the manufacturer. A PCR was conducted to target the ICP4 gene of ILTV using primers that were modified based on Chacon and Ferreira’s study [19]. DNA testing was performed on samples taken from chickens suspected of having ILT using specific primers: 5’-ACT GAT AGC TTT TCG TAC AGC ACG-3’ (forward primer) and 5’-CAT CGG GAC ATT CTC CAG GTA GCA-3’ (reverse primer). These primers aimed to amplify a particular 688-base pair (bp) section of the ICP4 gene. Each primer comprised 1 µl (10 pmol), nuclease-free water for 5.5 µl, DNA template for 5 µl, and 12.5 µl of Promega master mix, which made up the 25 µl PCR reaction mixture. A thermal cycler (Thermocycler, ASTEC, Japan) was used to conduct the PCR amplification. An initial denaturation step was performed at 94°C for 5 min. Then, there were 35 cycles of denaturation at 94°C for 1 min, annealing at 50°C for 30 sec, extension at 72°C for 1 min, and finally, an extension step at 72°C for 5 min, The PCR results were seen using electrophoresis on a 1.5% agarose gel in 1X TAE buffer. A solution of ethidium bromide with a concentration of 1 µg/ml was employed for the process of post-staining.

### Processing of samples for isolation of ILTV

Frozen ILT suspicious field samples were thawed and ground into a 10%–20% (w/v) solution using a sterile mortar and pestle in sterile 1 X PBS. The suspension was centrifuged for 10 min at 4°C and 5,000 rpm to provide clarity. After passing through a 0.22 µm Millipore filter, incubate the supernatant with antibiotics (2 mg/ml streptomycin, 50 µg/ml gentamycin, 2,000 units/penicillin, and 1,000 units/ml mycostatin) at room temperature for an hour. The inoculum was sterilely tested by putting it on a medium made of blood agar. The ILT virus was isolated using sterile inoculum on 9–10-day-old seronegative ECEs.

### Isolation of the ILT virus using seronegative ECEs

Five embryos have been designed by injecting 0.2 ml of inoculum (made from 10 PCR-positive samples) into each of the 9–10-day-old seronegative ECEs from a non-ILTV-vaccinated breeder farm. The embryos were then cultured at 37°C for 5–6 days. Both dead and alive embryos’ CAMs were inspected for ILTV-induced pock lesions, and allantoic fluid was collected for virus identification. Embryos were also checked for gross lesions such as hemorrhage, congestion, and edema. The hemagglutination test was conducted to identify hemagglutinating viruses such as NDV and AIV using 0.5% chicken red blood cells (cRBCs) on glass slides and observing for agglutination. Non-agglutinating samples were deemed negative for NDV and AIV and stored at −80°C. The positive samples underwent four consecutive passages in chicken embryos using the same method. The CAMs with clearly visible pock lesions were carefully collected, chopped into small pieces, and blended in a solution of 1X PBS to create a 10%–20% mixture. Afterward, the combination underwent centrifugation at a speed of 5,000 rpm for 20 min while being kept in refrigeration. The liquid portion was subsequently collected and preserved at a temperature of −80°C for further utilization.

### Identification of ILTV by PCR

The DNA was again extracted from allantoic fluid collected from ECEs and confirmed by PCR according to the above-described procedure.

### Nucleotide sequencing of the ICP4 gene and phylogenetic analysis

PCR products were used to investigate the nucleotide sequences of the ICP4 gene in recent ILTV isolates. The Neighbor-Joining approach was used to establish evolutionary links [20]. The evolutionary history of the studied species was represented by a bootstrap consensus tree, which was constructed using 1,000 repetitions. Branches that had a bootstrap support value of less than 50% were collapsed. The confidence levels of the bootstrap test, based on 1,000 repeats, are displayed next to the branches. To determine evolutionary distances, which are given as the number of base substitutions per site, the study employed the Maximum Composite Likelihood technique. A total of 31 nucleotide sequences were used in the study. For every sequence comparison, any unclear locations were eliminated using the pairwise deletion option. There were 570 locations in all that made up the dataset. The study conducted the evolutionary analyses using MEGA11 [21].

### Agar gel immunodiffusion test (AGIDT)

Following the protocol outlined by Barrow et al. [22], the study detected viruses in avian embryos taken from field samples using the AGID test. This test employed ILTV-specific hyper-immune serum. To prepare the agar gel, the study combined 1g of Agar Noble and 8 gm of sodium chloride in 100 ml of distilled water. The mixture was then microwaved for 2–3 min until melted and finally poured onto a glass slide to solidify at room temperature. Alim_ILT_1000 and Alim_ILT_1001, two ILTV field isolates, were cultured at room temperature for 1 h after being prepared with 1% NP40. Seven wells were created in the gel, with the standard anti-ILTV serum (GD Animal Health, Netherlands) placed in the central well and the two isolates, along with a known ILTV strain (HIPRAVIAR ILT vaccine) as a positive control and NDV, infectious bronchitis virus (IBV), and PBS as negative controls, in the peripheral wells. The gel was incubated at 8°C in a humid environment for 24 h, where a white precipitation line between antigen and antibody wells indicates a positive ILTV identification.

### Virus neutralization test (VNT)

VNTs were performed on 9–10-day-old seronegative ECEs via the CAM route. Serial tenfold dilutions of newly isolated viruses (10^−1^ to 10^−10^) were made in 1X PBS. The viral dilutions were mixed with a standard anti-ILTV serum at a ratio of 1:5 and then incubated for 1 h at room temperature. Afterward, 0.1 ml of each viral dilution and 0.2 ml of the serum-virus mixture were injected into the eggs through the CAM route, using five eggs for each dilution. After that, the eggs were incubated in a 37°C incubator for around 5 or 6 days. During this time, the eggs were examined twice a day using a candling technique, and any deaths that occurred were recorded daily. The Chicken Embryo Infective Dose 50 (CEID_50_) for both the virus and the serum-virus combination was determined using the Reed and Muench [23] technique. The neutralizing dosage of the serum was obtained by calculating the antilogarithm of the difference between the logarithm of the virus’s CEID_50_ (50% cell culture infectious dose) and the mixture of the serum and the virus.

### Pathogenicity tests of the ILTV isolates

The virulence of the ILT virus field isolates Alim_ILT_1001 and Alim_ILT_1000 was evaluated using the MICE method. In this method, a solution containing 10^3.0^ CEID_50_ of ILT virus was injected into 9–10-day-old ECEs using the CAM route, with a volume of 0.1 ml. Embryo mortality was checked twice daily for 7 days post-inoculation. The MICE was calculated as the proportion of viable embryos remaining after 7 days to the total number of deceased embryos during the same time.

The intratracheal pathogenicity index (ITPI) for the ILTV field was determined by assessing Alim_ILT_1001 and Alim_ILT_1000 using the approach established by El-Saied et al. [24] on 2–month–old seronegative layer chickens. The CEID_50_ for ILTV was calculated following Reed and Muench [23]. For the pathogenicity test, each of ten seronegative chickens was inoculated intratracheally with 0.1 ml of a 10^4.0^ CEID_50_ ILTV suspension, while ten control birds received 0.1 ml of 1X PBS. The clinical symptoms and mortality were observed and assessed for 14 days using the following scoring system: a score of 0 indicated a normal condition, a score of 1 indicated respiratory signs and a score of 2 indicated death. The total scores were divided by the product of the number of chickens and the observation period to determine the ITPI.

## Results

### Primary screening by NDV/AIV rapid test kit and ILTV by PCR

**Figure 2.**
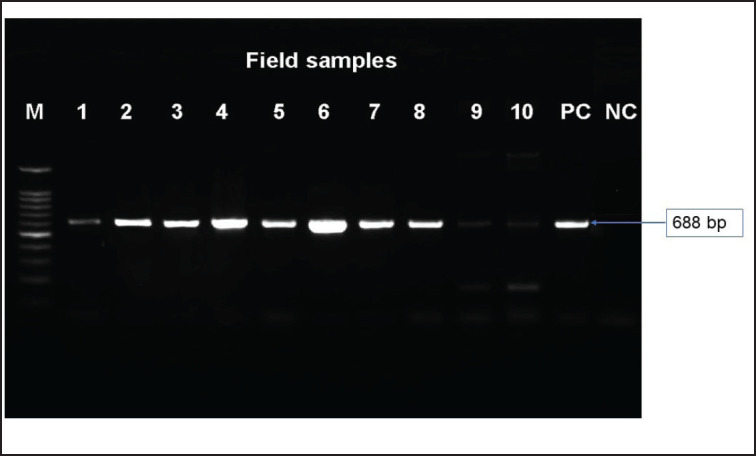
Rapid test kit (NDV/AIV) negative samples suspected for ILTV screening by PCR against the ICP4 gene. M = 100 bp DNA ladder, Lane 1–10 = positive field samples of ILTV; Lane PC = positive control and NC = negative control.

Out of the 345 samples, 182 were found to be positive for NDV, 58 for AIV, and 33 for a combination of the two infections. Among the 72 rapid test kit negative samples, only 10 were found positive by PCR using ILTV-specific primers, which were then used for the isolation and identification of the ILT virus (Fig. 2).

### Virus isolation using seronegative ECEs

**Figure 3.**
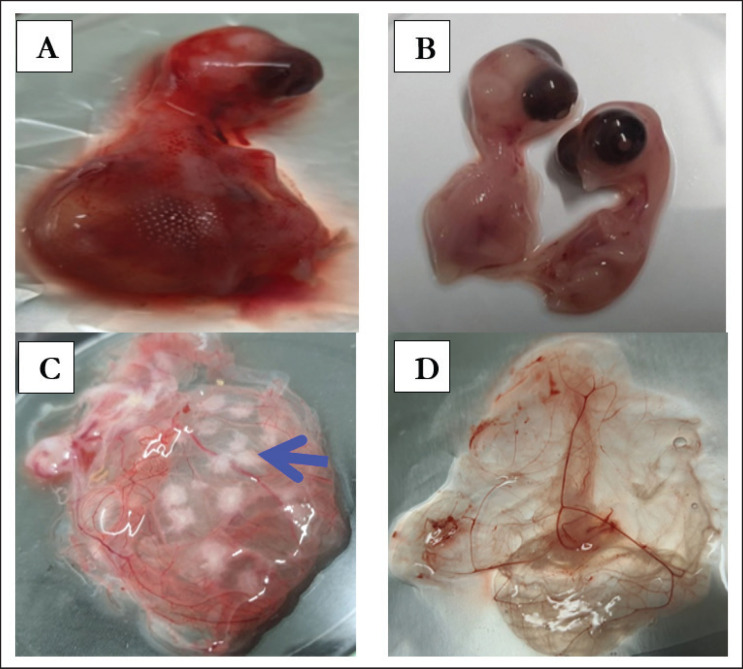
Isolation of ILTV using ECEs. A. Haemorrhagic and oedematous swelling of the ILTV-infected dead embryos; B. Control embryos; C. Infected CAM with ILTV revealed congestion and haemorrhage with characteristics of white pock lesions; D. Non-infected CAM inoculated with 1XPBS revealed normal membrane without any lesions**.**

A total of four serial passages were done for each sample for the isolation of the ILT virus. Embryo mortality usually starts from 48 to 60 h post-inoculation. The dead embryos showed edematous swelling and hemorrhagic lesions, while the alive embryos showed stunted growth in comparison to the control embryos (Fig. 3A and B). The pock lesions were evenly spread around the CAM and were seen to enlarge in consecutive passages progressively. The CAM lesions exhibited a range of sizes, ranging from scattered small foci to substantial concurrent lesions measuring up to 2–4 mm in diameter compared to the control (Fig. 3C and D). Thickening and edematous swelling were observed in the CAMs, along with the presence of white pock lesions.

### Reconfirmation of ILTV by PCR using the ICP4 gene

**Figure 4.**
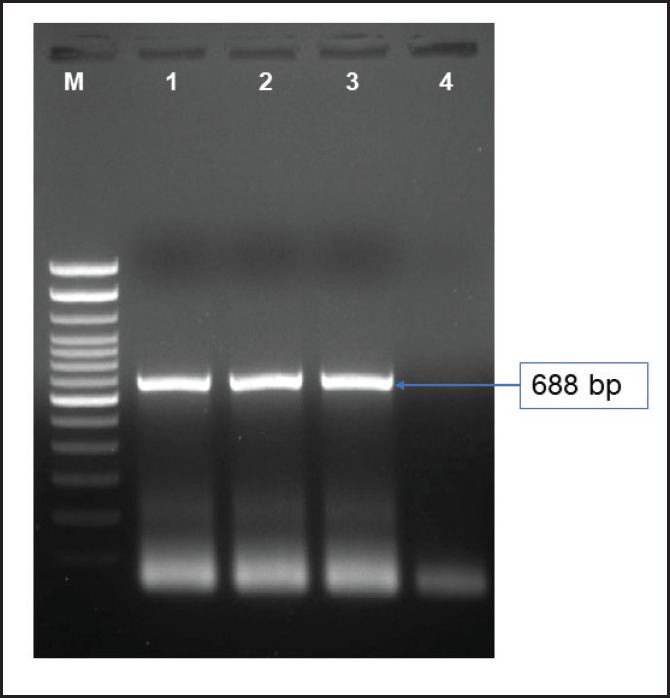
PCR amplification of ILTV isolates using primers against ICP4 gene. M = 100 bp DNA ladder, Lane 1 = positive control; Lane 2 and 3 = ILTV field isolates Alim_ILT_1001 and Alim_ILT_1000, and Lane 4 = Negative control.

Among the 10 ILTV-positive samples, only 2 were able to be isolated using ECEs, Alim_ILT_1001, and Alim_ILT_1000, as confirmed by PCR. These samples showed distinct bands at the expected size of 688 bp, indicating the presence of the ICP4 gene (Fig. 4).

**Figure 5.**
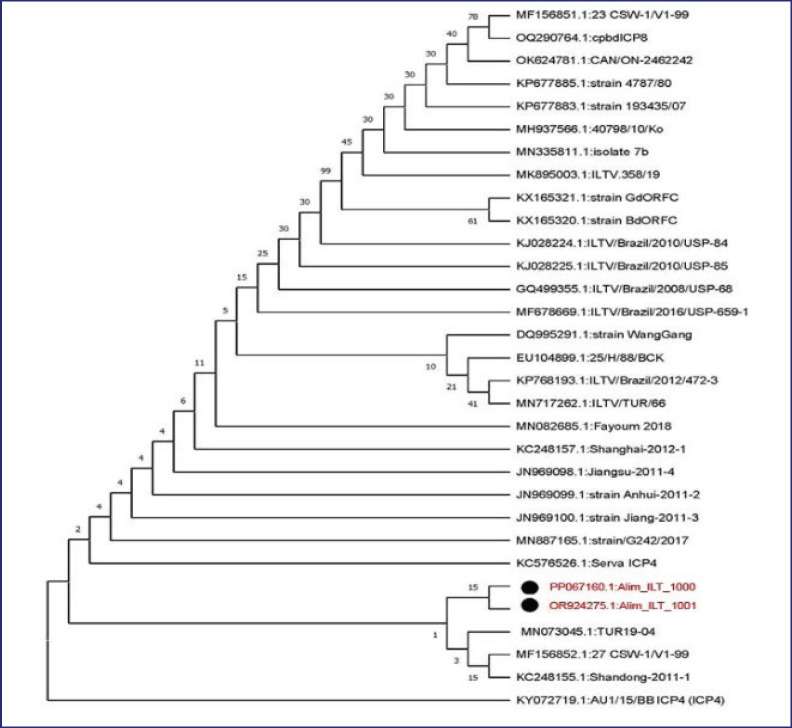
Phylogenetic analysis against the ICP4 gene of the recent isolates of ILTV by Neighbor-Joining method and the evolutionary investigations were carried out using the MEGA 11 program.

### Phylogenetic analysis of recently isolated ILTV

The phylogenetic study of the latest isolates of ILTV, using the partial nucleotide sequence of the ICP4 gene, shows a similarity to the isolates from Turkey, Bangladesh, and Australia (Fig. 5).

### Detection by serological tests

**Figure 6.**
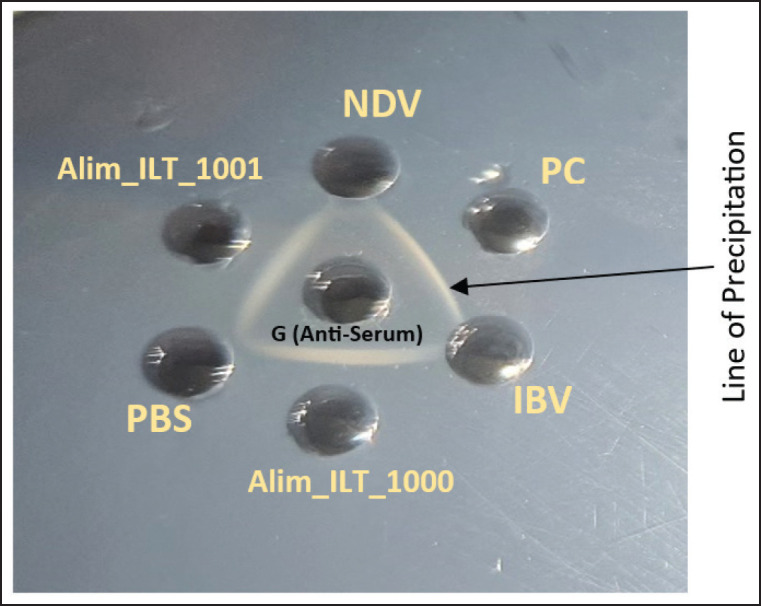
AGIDT of isolated field ILTV. Central well (G) contains ILTV-specific anti-serum against ILTV. Periphery wells contain negative control (NDV, IBV, and PBS), positive control (live HIPRAVIAR ILT vaccine), and two ILTV filed isolates Alim_ILT_1001 and Alim_ILT_1000.

The AGID test revealed that two ILTV field samples, Alim_ILT_1001 and Alim_ILT_1000, were positive for the ILT virus and exhibited a strong line of precipitation (Fig. 6). Both the isolated ILTVs were neutralized by ILTV-specific anti-serums. The NI (neutralization index) to log_10_CEID_50 _with anti-serum against ILTV ranged from 5.18 to 5.35, and the mean NI was 5.27 (Table 1).

### Pathogenicity tests of isolated ILTV

The control group had a MICE of 0.00, but the infected group had a MICE value of 0.77 for the Alim_ILT_1001 field isolate and 0.71 for the Alim_ILT_1000 field isolate (Table 2). The control group had an ITPI value of 0.00, but the infected group had an ITPI value of 0.63 for the Alim_ILT_1001 field isolate and 0.57 for the Alim_ILT_1000 field isolate (Table 3).

**Table 1.** Results of virus neutralization test of ILT virus isolates using specific anti serum against ILTV.

Isolated ILTV isolates	Test component	No. of infected CAM at different dilution of virus and virus/anti-serum mixture										Log_10_ CEID_50_/ml titer	NI
		10−^1^	10−^2^	10−^3^	10−^4^	10−^5^	10−^6^	10−^7^	10−^8^	10−^9^	10−^10^		
Alim_ILT_1001	Virus	5/5	5/5	5/5	5/5	5/5	4/5	3/5	2/5	1/5	0/5	8.5	5.18
	Virus/anti-serum	5/5	3/5	1/5	0/5	0/5	0/5	0/5	0/5	0/5	0/5	3.32	
Alim_ILT_1000	Virus	5/5	5/5	5/5	5/5	5/5	3/5	2/5	1/5	0/5	0/5	7.67	5.35
	Virus/anti-serum	3/5	1/5	0/5	0/5	0/5	0/5	0/5	0/5	0/5	0/5	2.32	

Prefix = No. of embryos infected, Suffix = No. of total embryos, NI = Neutralization index, CEID_50_ = Chicken embryo infective dose 50, ILT = Infectious laryngotracheitis.

**Table 2.** Determination of MICE into seronegative ECEs.

Isolated ILTV isolates	Status of ECEs	% of mortality of chicken embryos on day of post inoculation							Cumulative Number of ECEs	MICE
Day-1	Day-2	Day-3	Day-4	Day-5	Day-6	Day-7
Alim_ILT_1001	Dead	0	1	6	10	13	15	16	61	0.77*
	Alive	20	19	14	10	7	5	4	79	
Alim_ILT_1000	Dead	0	1	6	10	12	14	15	58	0.71*
	Alive	20	19	14	10	8	6	5	82	
PBS Control	Dead	0	0	0	0	0	0	0	0.00	0.00
	Alive	20	20	20	20	20	20	20	140	

ILTV- Infectious laryngotracheitis virus; ECEs- Embryonated chicken eggs; MICE- mortality index of chicken embryo; PBS- Phosphate buffered saline.

* indicates the MICE value more than 0.27 is considered as highly pathogenic for chickens.

**Table 3.** Determination of ITPI using layer chickens.

Isolated ILTV isolates	Clinical Signs	D-1	D-2	D-3	D-4	D-5	D-6	D-7	D-8	D-9	D-10	D-11	D-12	D-13	D-14	Total	Score	ITPI Value
Alim_ILT_1001	Normal	10	10	10	9	7	6	5	3	0	0	0	0	0	0	60×0	0.0	0.63*
	Sick	0	0	0	1	3	4	5	7	10	9	9	8	8	8	72×1	72	
	Dead	0	0	0	0	0	0	0	0	0	1	1	2	2	2	08×2	16	
	Total =																88	
Alim_ILT_1000	Normal	10	10	10	9	8	7	6	5	3	0	0	0	0	0	68×0	0.0	0.57*
	Sick	0	0	0	1	2	3	4	5	7	9	9	8	8	8	64×1	64	
	Dead	0	0	0	0	0	0	0	0	0	1	1	2	2	2	08×2	16	
	Total =																80	
PBS Control	Normal	10	10	10	10	10	10	10	10	10	10	10	10	10	10	140×0	0.0	0.00
	Sick	0	0	0	0	0	0	0	0	0	0	0	0	0	0	0×1	0.0	
	Dead	0	0	0	0	0	0	0	0	0	0	0	0	0	0	0×2	0.0	
	Total=																0.0	

ILTV- Infectious laryngotracheitis virus; ITPI-Intra-tracheal pathogenicity index; PBS- Phosphate buffered saline.

* indicates ITPI value ranges from 0.20-0.82 is considered as virulent strains for chickens.

## Discussion

In recent years, outbreaks of respiratory viral diseases with variable mortality rates have increased in the chicken population. Although ILT disease kills fewer birds than AI or ND, it significantly reduces the productivity of the surviving birds. Sensitive, specific, and rapid tests are required to identify diseased flocks, minimize disease transmission to susceptible flocks, avoid excessive treatment costs, and encourage vaccination in huge populations. In the present study, the isolation and characterization of ILTV were performed.

Zorman et al. [7] and Kirkpatrick et al. [25] found that clinical signs of ILTV infection include tracheal rales, severe tracheitis, gasping, expectoration of bloody mucus, decreased egg production, low mortality, swelling of the infra-orbital sinuses, and conjunctivitis. Postmortem (PM) findings include coughed-up blood, tracheal blood clots, hemorrhagic lesions in the trachea and larynx, as well as edema and congestion of the infra-orbital sinuses. Similar types of clinical signs and PM findings were observed in the present study during sample collection from ILT-suspected birds.

Among 10 ILTV suspected field samples, only two (Alim_ILT_1001 and Alim_ILT_1000) produced distinct pock lesions when inoculated through the CAM route of 9–10-day-old seronegative ECEs. The changes in embryo and CAM produced by suspected ILTV have similarities with the findings explained by Islam et al. [13], Gowthaman et al. [2], and Kammon et al. [26].

In this study, the isolation rate of ILTV from 10 PCR-screening-positive field samples was found to be relatively lower because the live virus in the collected samples might have been inactivated during the collection and processing of the samples. It might be due to the similarity in clinical manifestations caused by other respiratory viruses such as NDV, AIV, and IBV. This often leads to challenges in accurately diagnosing ILTV based solely on clinical signs.

The PCR results in this study revealed that two field isolates of the ILTV exhibited distinct bands at the expected size of 688 base pairs, corresponding to the ICP4 gene. Razmyar et al. [27] and Chacon et al. [19] also used a similar gene to detect ILTV from field samples by PCR, which supports our present finding. The phylogenetic study constructed on the ICP4 gene of both isolates showed a close genetic relationship with isolates from Turkey, Bangladesh, and Australia. This indicates a possible genetic similarity or common ancestry between these isolates. This suggests that the ILTV strains from different geographical regions might share significant genetic characteristics, particularly in the ICP4 gene region. The study agrees with the research conducted by Kalin et al. [28] and Yang et al. [29]. Additionally, both strains showed a close relationship in nucleic acid sequences of the ICP4 to other reference strains that have been isolated from various countries in Asia and a homology between 99.77% and 100% [28,29].

Serological identification by AGDT and VNT of both the field isolates was also performed. These results are parallel to the findings of Islam et al. [13] and Fukui et al. [30], who also identified ILTV through the AGID test, PHA test, and VNT test.

The pathogenicity of isolated ILTV using the titration method indicated that the CAM suspension contained a higher virus load than allantoic fluid and embryo. The result of the pathogenicity tests showed the MICE values of Alim_ILT_1001 and Alim_ILT_1000 isolates were 0.77 and 0.71, respectively. The result aligns with the research conducted by Yan et al. [31], which indicated that MICE over 0.27 is classified as very harmful for chickens. On the other hand, the ITPI values for Alim_ILT_1001 and Alim_ILT_1000 isolates were 0.63 and 0.57, respectively, which indicated that the field isolates of ILTV are highly virulent strains for chickens, according to the report of Shehata et al. [32], who found that the ITPI value ranges from 0.20 to 0.82 and is considered a virulent strain for chickens.

## Conclusion

ILT continues to be a significant economic burden in Bangladesh’s poultry industry. The recently isolated field strains of ILTV were found to be virulent for chickens, according to pathogenicity tests. ICP4 gene-based phylogenetic analysis of the recent isolates indicates that the circulating strains of ILTV have a close similarity with the isolates of Turkey, Bangladesh, and Australia, which suggests that the ILTV circulating among the layer chicken population of Bangladesh might have been introduced from those two countries either through vaccination or evolved internally through mutation. To eradicate ILT, it is necessary to substitute modified live ILT vaccinations with inactivated vaccines derived from recent isolates. This measure is crucial to prevent the virus from proliferating and increasing its contagiousness.

## List of Abbreviations

AIV, avian influenza virus; AGIDT, agar gel immunodiffusion test; CAM, chorioallantoic membrane; cRBC, chicken red blood cells; DNA, deoxyribonucleic acid; ECEs, embryonated chicken eggs; ELISA, enzyme linked immunosorbent assay; IBV, infectious bronchitis virus;

ILTV, infectious laryngotracheitis virus; ILT, infectious laryngotracheitis; ITPI, intra-tracheal pathogenicity index; MICE, mortality index for chicken embryos; NDV, Newcastle diseases virus; PCR, polymerase chain reaction; PHA, passive hemagglutination; VNT, virus neutralization test
